# A Case of Cold Agglutinin Disease With Transformation to High-Grade Lymphoma During Sutimlimab Treatment

**DOI:** 10.7759/cureus.87006

**Published:** 2025-06-29

**Authors:** Shota Yamaguchi, Kenki Saito, Keiji Shimada, Naoya Kaneko

**Affiliations:** 1 Hematology and Oncology, Nara City Hospital, Nara, JPN; 2 Hematology and Oncology, Nara Prefecture General Medical Center, Nara, JPN; 3 Blood Transfusion Medicine, Nara Medical University, Nara, JPN; 4 Pathology, Nara City Hospital, Nara, JPN; 5 Hematology, Nerima Hikarigaoka Hospital, Nara, JPN

**Keywords:** aggressive lymphoma, autoimmune-hemolytic anemia, b-cell lymphoma, bone marrow biopsy, cold agglutinin disease, p-r-chp therapy, sutimlimab

## Abstract

Cold agglutinin disease (CAD) is an autoimmune hemolytic anemia characterized by monoclonal immunoglobulin M-mediated cold agglutinins and monoclonal B-cell proliferation. Sutimlimab, a complement C1s inhibitor, alleviates hemolytic anemia in CAD by blocking the classical complement pathway. We report a case of a 67-year-old Japanese woman with CAD who experienced temporal improvement in anemia following sutimlimab treatment. ^18^F-fluorodeoxyglucose positron-emission tomography/computed tomography and bone marrow biopsy (BMB) revealed histological transformation into aggressive B-cell lymphoma. Subsequent chemotherapy with polatuzumab vedotin, rituximab, cyclophosphamide, doxorubicin, and prednisolone resulted in further improvement in anemia. This case highlights the importance of reassessing underlying conditions through BMB in cases where sutimlimab treatment is ineffective.

## Introduction

Primary cold agglutinin disease (CAD) is a rare form of autoimmune hemolytic anemia caused by monoclonal immunoglobulin M (IgM)-mediated cold agglutinins and monoclonal B-cell proliferation. CAD occasionally presents with false macrocytosis, in which the mean corpuscular volume (MCV) may appear falsely elevated due to autoagglutination of red blood cells. CAD was first included in the fifth edition of the World Health Organization Classification of Tumors [1]. In contrast, CAD secondary to an underlying condition is referred to as cold agglutinin syndrome (CAS), which is considered a distinct clinical entity from CAD. CAD typically follows an indolent course and has a generally favorable prognosis. Transformation into aggressive lymphoma has been reported in approximately 3.5% of patients during a 10-year follow-up period [2]. CAD management primarily involves avoiding cold exposure; however, no standardized treatment has been established for patients with moderate to severe disease who do not respond to cold avoidance alone.

Sutimlimab, a monoclonal antibody targeting complement component C1s, has recently emerged as a treatment option for CAD. It improves hemolytic anemia in CAD by inhibiting the classical complement pathway, which is the main mechanism underlying hemolysis in this condition [3]. In the phase 3 CARDINAL trial, which enrolled patients with CAD and a history of transfusion, sutimlimab was shown to increase hemoglobin (Hb) levels and reduce fatigue [4]. Based on these findings, sutimlimab was approved for the treatment of CAD in Japan in June 2022. Herein, we report a case of a Japanese woman with CAD associated with a B-cell lymphoproliferative disorder (B-LPD), who experienced only a temporary improvement in anemia following sutimlimab treatment. Further evaluations revealed histological transformation to aggressive B-cell lymphoma. Subsequent chemotherapy resulted in improvement of the anemia.

## Case presentation

A 67-year-old Japanese woman with a history of hypertension presented to a local clinic three weeks prior to visiting our institution, complaining of epigastric abdominal pain and exertional fatigue. Laboratory tests revealed macrocytic anemia, with an Hb level of 7.8 g/dL and an MCV of 110 fL. Levels of vitamin B12, folic acid, thyroid hormones, and copper were all within normal limits. Upper gastrointestinal endoscopy showed no bleeding or space-occupying lesions that could explain the anemia. Subsequently, she was referred to our hematology department for further examination and treatment. Laboratory test results at the initial visit are summarized in Table 1. Findings indicated hemolytic anemia with an Hb level of 7.5 g/dL, reticulocyte count of 21.64 × 10⁴/μL, lactate dehydrogenase (LDH) of 355 U/mL, total bilirubin of 1.97 mg/dL (indirect bilirubin 1.42 mg/dL), and haptoglobin level <10 mg/dL.

**Table 1:** Laboratory data of the patient at the initial visit WBC: white blood cell count; RBC: red blood cell count; Hb: hemoglobin; Plt: platelets; PT-INR: prothrombin time-international normalized ratio; APTT: activated partial thromboplastin time; Fbg: fibrinogen; FDP: fibrinogen degradation products; TP: total protein; Alb: albumin; AST: aspartate transaminase; ALT: alanine transaminase; LDH: lactate dehydrogenase; T-Bil: total bilirubin; D-Bil: direct bilirubin; BUN: blood urea nitrogen; Cre: creatinine; eGFR: estimated glomerular filtration rate; UA: uric acid; Zn: zinc; TSH: thyroid-stimulating hormone; FreeT4: free thyroxine; Fe: serum iron; TIBC: total iron-binding capacity; UIBC: unsaturated iron-binding capacity; IgG: immunoglobulin G; IgA: immunoglobulin A; IgM: immunoglobulin M

Complete blood count	Result	Normal range
WBC (/μL)	4190	3300-8600
RBC (×10⁴/μL)	224	386-492
Hb (g/dL)	7.5	11.6-14.8
Plt (×10⁴/μL)	21.3	15.8-34.8
Reticulocyte (%)	9.7	0.3-1.5
Absolute reticulocyte count (×10⁴/μL)	21.64	3.0-8.0
PT-INR	1.05	0.9-1.2
APTT (sec)	23.4	25-40
Fbg (mg/dL)	340	180-340
FDP (μg/mL)	＜2.5	0-5.0
Direct Coombs test	(+)	N/A
Indirect Coombs test	(-)	N/A
Anti-human IgG antibody	(-)	N/A
Anti-complementary antibody	2+	N/A
Irregular antibodies	(-)	N/A
TP (g/dL)	7.4	6.6-8.1
ALB (g/dL)	4.3	4.1-5.1
AST (U/L)	23	13-30
ALT (U/L)	18	7-23
LDH (U/L)	355	124-222
T-Bil (mg/dL)	1.97	0.4-1.5
D-Bil (mg/dL)	0.55	0-0.4
BUN (mg/dL)	19.6	8.0-20.0
Cre (mg/dL)	0.77	0.46-0.79
eGFR (mL/min/1.73㎡)	57.3	N/A
UA (mg/dL)	6.8	2.6-5.5
Zn (μg/dL)	80	80-130
TSH (μIU/mL)	2.34	0.5-5.0
FreeT4 (ng/dL)	1.04	0.9-1.7
Erythropoietin (mIU/mL)	181	4.2-23.7
Fe (μg/dL)	158	43-172
TIBC (μg/dL)	315	251-398
UIBC (μg/dL)	157	137-325
Ferritin (ng/mL)	335	12-60
Haptoglobin (mg/dL)	10>	N/A
IgG (mg/dL)	891.8	851-1747
IgA (mg/dL)	150.3	93-393
IgM (mg/dL)	1540.1	50-269
Vitamin B12 (pg/mL)	603	180-914
Folic acid (ng/mL)	9.2	>4.0
Serum Copper (µg/dL)	166	68-128

**Figure 1:**
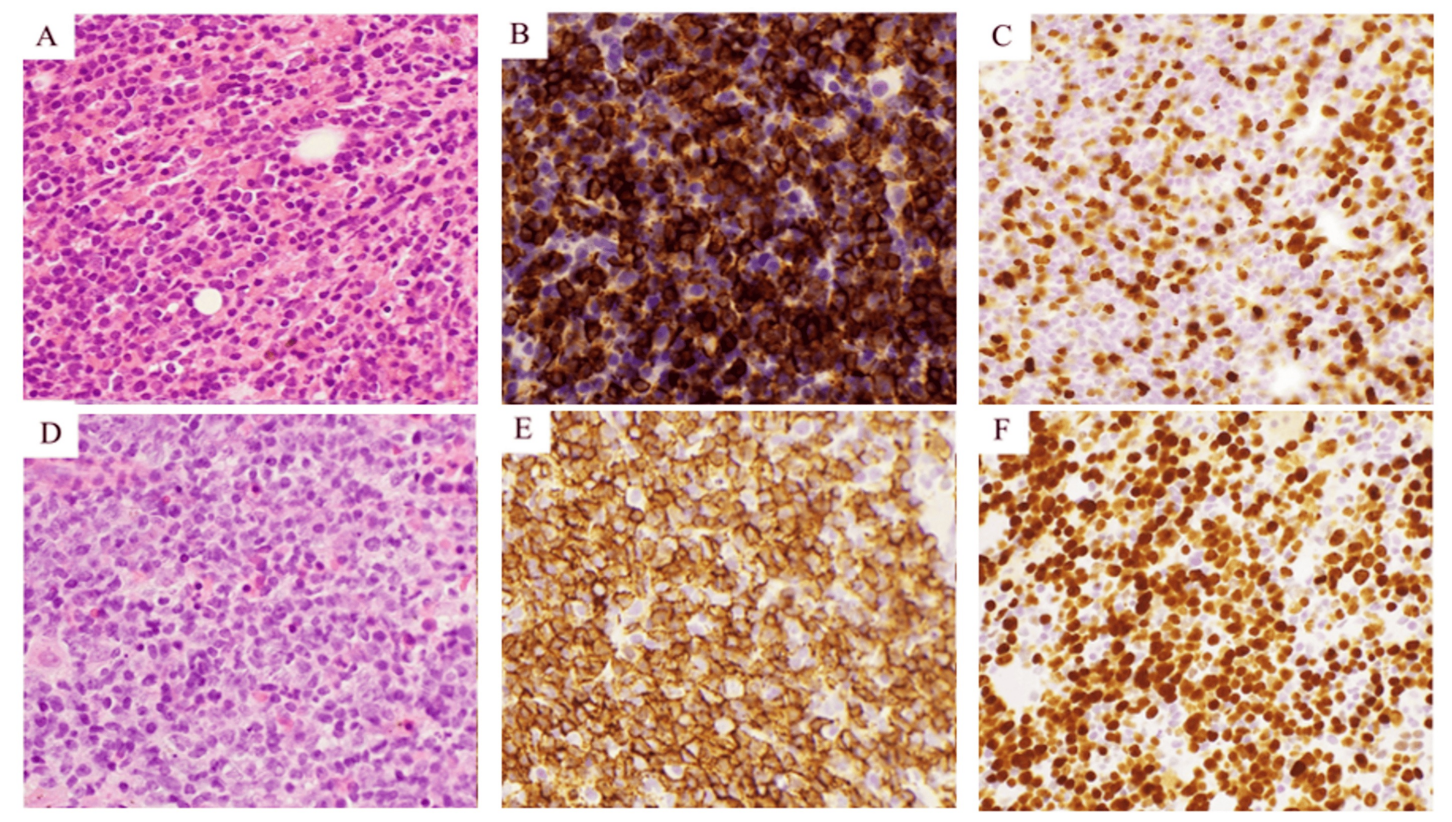
Pathologic findings of the bone marrow biopsies at onset (A-C) and exacerbation (D-F) BMB at onset revealed nodular or interstitial diffuse growth of small- to medium-sized B-cells (A, H&E, ×200) and B-cells positive for CD20 (B, H&E, ×200). BMB at exacerbation revealed sheets of large cells with centroblastic, immunoblastic, or anaplastic morphology and brisk mitotic figures (D, H&E, ×200) and cells positive for CD20 (E, H&E, ×200). The Ki67-labeling index was 50-60% (F, H&E, ×200), higher than that at onset (C, H&E, ×200). H&E: hematoxylin and eosin; BMB: bone marrow biopsy

**Figure 2:**
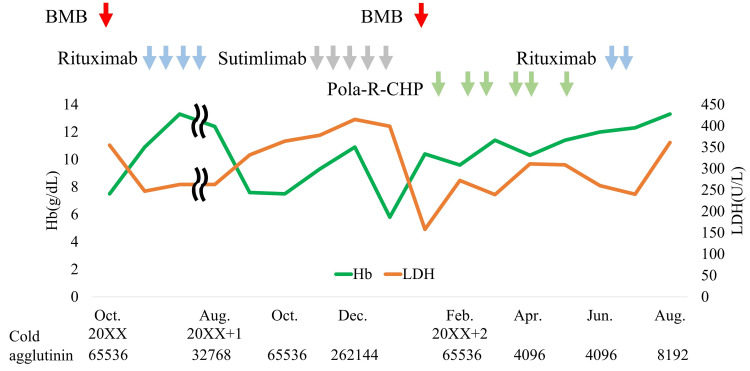
Overview of treatment and laboratory findings in the patient BMB: bone marrow biopsy; Hb: hemoglobin; LDH: lactate dehydrogenase; P-R-CHP: polatuzumab vedotin, rituximab, cyclophosphamide, doxorubicin, and prednisolone

Furthermore, the anti-complement direct Coombs test was strongly positive. IgM was elevated at 1540 mg/dL, and the cold agglutinin titer was markedly high at 65,536. Contrast-enhanced computed tomography (CT) revealed no apparent lymphadenopathy or splenomegaly. A bone marrow biopsy (BMB) showed a total nucleated cell count of 2.3 × 10⁵/μL, with lymphocytes comprising 9.8% of the marrow cells. Nodular or interstitial diffuse proliferation of CD20-positive small- to medium-sized B-cells was observed (Figure 1A-1B), and immunoglobulin light chains demonstrated kappa chain restriction. The Ki67 labeling index was 50-60% (Figure 1C). Features characteristic of lymphoplasmacytic lymphoma, such as paratrabecular growth, fibrosis, lymphoplasmacytoid cell morphology, and mast cell accumulation around lymphoid aggregates, were absent. Based on these findings, a provisional diagnosis of CAD associated with B-LPD was made (Figure 2).

**Figure 3:**
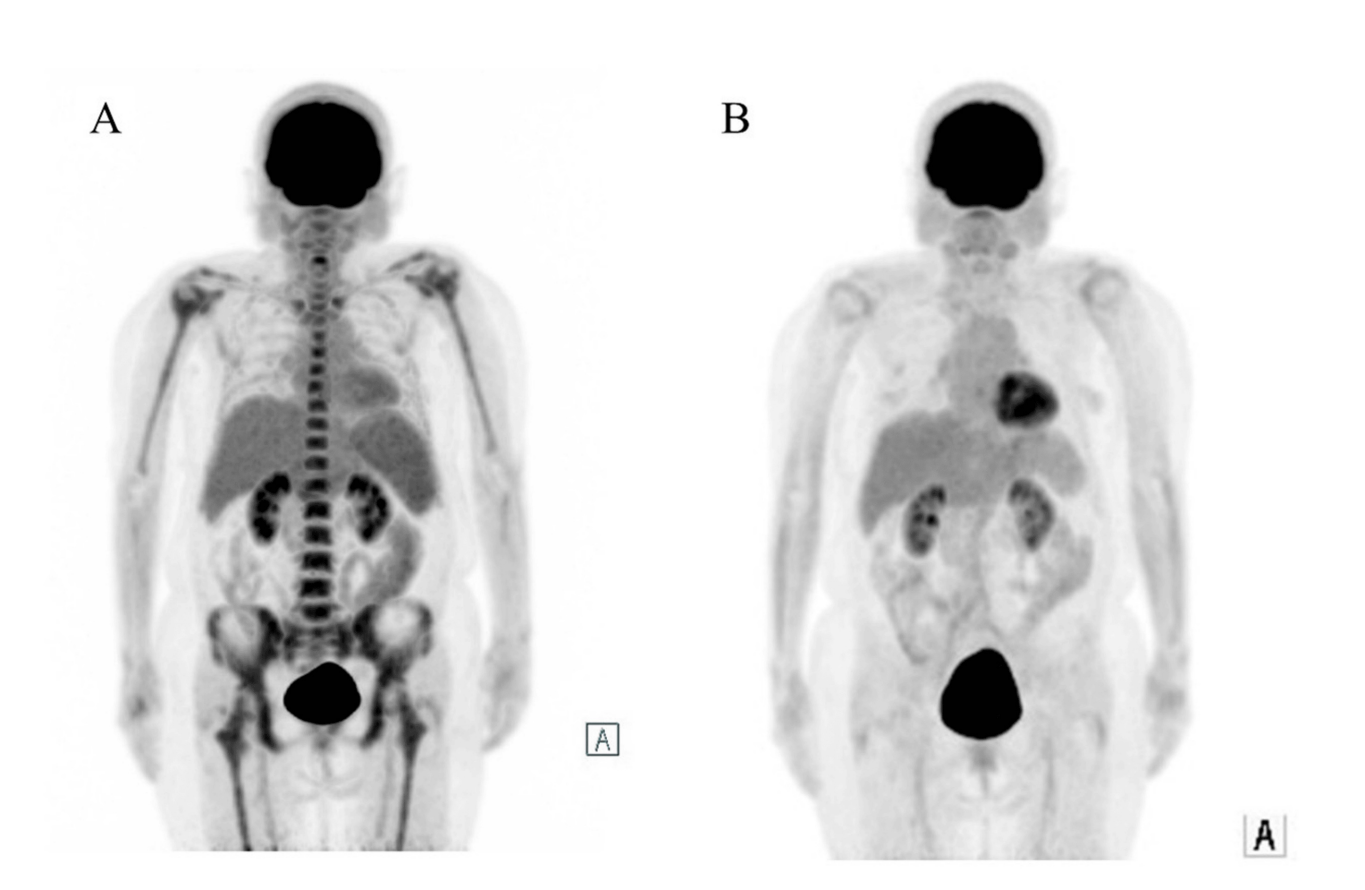
Comparison of 18F-FDG PET-CT images before and after chemotherapy (A) Diffuse ^18^F-FDG uptake in the bone marrow and increased ^18^F-FDG uptake in the spleen were seen at disease exacerbation before starting chemotherapy. (B) No ^18^F-FDG uptake suggestive of lymphoma lesions was observed in the bone marrow, spleen, or lymph nodes after chemotherapy, which is consistent with a complete metabolic response. ^18^F-FDG PET-CT: ^18^F-fluorodeoxyglucose positron emission tomography/computed tomography

Treatment with sutimlimab was initiated at 6.5 mg/kg. The second dose was administered one week later, followed by dosing every two weeks thereafter. Anemia improved, and Hb increased to 10.9 g/dL after the third dose. However, Hb decreased to 7.7 g/dL after the fourth dose. In addition, elevated levels of LDH (355 U/mL), IgM (3301.7 mg/dL), and soluble interleukin-2 receptor (5956 U/mL) suggested transformation to aggressive B-cell lymphoma. ^18^F-fluorodeoxyglucose positron emission tomography/computed tomography (^18^F-FDG PET-CT) showed splenomegaly with increased ^18^F-FDG uptake (maximum standardized uptake value (SUVmax) = 3.2) and diffuse ^18^F-FDG uptake in the bone marrow (SUVmax = 5.5), consistent with malignant lymphoma (Figure 3A). BMB revealed sheets of CD20-positive large cells with centroblastic, immunoblastic, or anaplastic morphology, along with brisk mitotic activity (Figures 1D-1E). The Ki67 labeling index was 50-60% (Figure 1F), consistent with histological transformation to aggressive B-cell lymphoma. Sutimlimab treatment was discontinued after five doses, and chemotherapy with polatuzumab vedotin combined with rituximab, cyclophosphamide, doxorubicin, and prednisolone (Pola-R-CHP) was initiated. On day 2 of treatment, the Hb level dropped to 6.3 g/dL, necessitating the transfusion of two units of red blood cells. Subsequently, anemia improved, and no further transfusions were required. After six courses of Pola-R-CHP and two additional doses of rituximab, ^18^F-FDG PET-CT demonstrated metabolic complete remission (mCR) (Figure 3B).

## Discussion

We report a case of disease exacerbation occurring during sutimlimab treatment for CAD. Although the initial bone marrow examination revealed B-LPD, a subsequent BMB performed during sutimlimab treatment revealed features of high-grade B-cell lymphoma, suggesting histological transformation. Chemotherapy was administered for the underlying lymphoma, and the patient has remained stable without recurrence of either lymphoma or anemia. CAD typically progresses slowly. A population-based retrospective study involving 86 patients with CAD reported a median survival of 12.5 years (median age at diagnosis: 67 years) [2]. Similarly, an international collaborative retrospective study by Berentsen et al. estimated a median survival time of 16 years, with a five-year survival rate of 83% [5]. During the eight-year follow-up period of that study, only eight cases (3.4%) of diffuse large B-cell lymphoma were observed, indicating a low incidence of transformation. The anti-CD20 antibody rituximab has been the primary treatment for moderate to severe CAD unresponsive to cold avoidance. In a small prospective trial, rituximab monotherapy achieved a response rate of approximately 50%, with a mean Hb increase of 4.0 g/dL from baseline and a median response duration of less than one year, indicating its limited efficacy [6]. By contrast, combination therapy with rituximab and bendamustine has been used to further reduce the underlying B-cells. Berentsen et al. conducted a prospective, non-controlled, multicenter trial involving 45 patients with CAD. They reported that combination treatment with rituximab and bendamustine achieved a partial or better response in approximately 70% of cases [7]. However, these treatments are currently considered off-label in Japan.

Sutimlimab, a monoclonal antibody targeting C1s, improves anemia by selectively inhibiting the upstream processes of the classical complement pathway. In the CARDINAL trial, clinically significant improvements in anemia and fatigue were observed within one week of initiating sutimlimab, with Hb levels maintained during treatment [4]. While sutimlimab is effective in CAD, its efficacy in CAS, a secondary disorder associated with infections or lymphoid tumors such as chronic lymphocytic leukemia, has not been confirmed. For CAS, treatment of the underlying condition is recommended; thus, assessment of underlying diseases is essential for distinguishing between CAD and CAS. In this case report, the initial bone marrow findings of B-LPD were considered consistent with CAD. The patient's Hb levels improved after initial treatment with rituximab and remained stable for 10 months after discontinuation, suggesting slow disease progression. However, upon relapse, the effect of sutimlimab was only temporary, unlike the findings from clinical trials. Consequently, a repeat bone marrow examination was conducted to reassess the disease, which revealed histological transformation to aggressive B-cell lymphoma. Subsequent chemotherapy targeting the aggressive lymphoma led to improvements in both lymphoma and anemia. After completing Pola-R-CHP, ^18^F-FDG PET-CT findings indicated mCR, with no subsequent decline in Hb levels. Given that sutimlimab does not reduce the B-cell clones that cause CAD, treatment targeting aggressive lymphoma is necessary in cases of histological transformation, as seen in our case study. However, even in CAS, as in this case, sutimlimab administration may temporarily improve anemia, potentially delaying the detection of disease progression, including histological transformation. Although CAD transformation is rare, careful monitoring for disease progression during sutimlimab treatment remains crucial.

## Conclusions

CAD generally follows an indolent course; however, histological transformation, although rare, can occur. In our case, sutimlimab temporarily improved anemia; however, it subsequently worsened. A repeat BMB revealed transformation to aggressive lymphoma. Chemotherapy targeting the lymphoma successfully improved anemia and achieved an mCR. These observations underscore the need for reassessment with repeat BMB when sutimlimab treatment is ineffective.
